# Expansion-enhanced super-resolution radial fluctuations enable nanoscale molecular profiling of pathology specimens

**DOI:** 10.1038/s41565-023-01328-z

**Published:** 2023-04-10

**Authors:** Dominik Kylies, Marina Zimmermann, Fabian Haas, Maria Schwerk, Malte Kuehl, Michael Brehler, Jan Czogalla, Lola C. Hernandez, Leonie Konczalla, Yusuke Okabayashi, Julia Menzel, Ilka Edenhofer, Sam Mezher, Hande Aypek, Bernhard Dumoulin, Hui Wu, Smilla Hofmann, Oliver Kretz, Nicola Wanner, Nicola M. Tomas, Susanne Krasemann, Markus Glatzel, Christoph Kuppe, Rafael Kramann, Bella Banjanin, Rebekka K. Schneider, Christopher Urbschat, Petra Arck, Nicola Gagliani, Marc van Zandvoort, Thorsten Wiech, Florian Grahammer, Pablo J. Sáez, Milagros N. Wong, Stefan Bonn, Tobias B. Huber, Victor G. Puelles

**Affiliations:** 1 III. Department of Medicine, University Medical Center Hamburg-Eppendorf, Hamburg, Germany; 2 Hamburg Center for Kidney Health (HCKH), University Medical Center Hamburg-Eppendorf, Hamburg, Germany; 3 Research Center On Rare Kidney Diseases (RECORD), University Hospital Erlangen, Erlangen, Germany; 4 Institute of Medical Systems Biology, Center for Biomedical AI (bAIome), Center for Molecular Neurobiology Hamburg (ZMNH), University Medical Center Hamburg-Eppendorf, Hamburg, Germany; 5 Cell Communication and Migration Laboratory, Department of Biochemistry and Molecular Cell Biology (IBMZ), Center for Experimental Medicine, Hamburg, Germany; 6 Department of General, Visceral and Thoracic Surgery, University Medical Center Hamburg-Eppendorf, Hamburg, Germany; 7 Mildred Scheel Cancer Career Center HaTriCS4, University Medical Center Hamburg-Eppendorf, Hamburg, Germany; 8 Division of Nephrology and Hypertension, Department of Internal Medicine, The Jikei University School of Medicine, Tokyo, Japan; 9 Abberior Instruments GmbH, Göttingen, Germany; 10 Institute of Neuropathology, University Medical Center Hamburg-Eppendorf, Hamburg, Germany; 11 Institute of Experimental Medicine and Systems Biology and Division of Nephrology and Clinical Immunology, RWTH Aachen University Medical Faculty, Aachen, Germany; 12 Department of Developmental Biology, Erasmus Medical Center, Rotterdam, The Netherlands; 13 Oncode Institute, Erasmus Medical Center Cancer Institute, Rotterdam, The Netherlands; 14 Institute for Cell and Tumor Biology, RWTH Aachen University, Aachen, Germany; 15 Department of Obstetrics and Fetal Medicine, Division of Experimental Feto-Maternal Medicine, University Medical Center Hamburg-Eppendorf, Hamburg, Germany; 16 I. Department of Medicine, University Medical Center Hamburg-Eppendorf, Hamburg, Germany; 17 Department of Genetics and Cell Biology, Maastricht University, School for Oncology and Reproduction GROW, School for Mental Health and Neuroscience MHeNS, and School for Cardiovascular Diseases CARIM, Maastricht University, Maastricht, The Netherlands; 18 Institute for Molecular Cardiovascular Research (IMCAR), RWTH Aachen University, Aachen, Germany; 19 Institute of Pathology, University Medical Center Hamburg-Eppendorf, Hamburg, Germany; 20 Department of Clinical Medicine, Aarhus University, Aarhus, Denmark; 21 Department of Pathology, Aarhus University Hospital, Aarhus, Denmark

**Keywords:** Nanostructures, Super-resolution microscopy

## Abstract

Expansion microscopy physically enlarges biological specimens to achieve nanoscale resolution using diffraction-limited microscopy systems^1^. However, optimal performance is usually reached using laser-based systems (for example, confocal microscopy), restricting its broad applicability in clinical pathology, as most centres have access only to light-emitting diode (LED)-based widefield systems. As a possible alternative, a computational method for image resolution enhancement, namely, super-resolution radial fluctuations (SRRF)^2,3^, has recently been developed. However, this method has not been explored in pathology specimens to date, because on its own, it does not achieve sufficient resolution for routine clinical use. Here, we report expansion-enhanced super-resolution radial fluctuations (ExSRRF), a simple, robust, scalable and accessible workflow that provides a resolution of up to 25 nm using LED-based widefield microscopy. ExSRRF enables molecular profiling of subcellular structures from archival formalin-fixed paraffin-embedded tissues in complex clinical and experimental specimens, including ischaemic, degenerative, neoplastic, genetic and immune-mediated disorders. Furthermore, as examples of its potential application to experimental and clinical pathology, we show that ExSRRF can be used to identify and quantify classical features of endoplasmic reticulum stress in the murine ischaemic kidney and diagnostic ultrastructural features in human kidney biopsies.

## Abstract (web summary)

Complex clinical samples can be imaged with 25 nm resolution using a light-emitting-diode-based wide-field microscope.

## Main

Super-resolution microscopy refers to a group of methodologies that break the barrier of the diffraction limit, a physical obstacle restricting the optical resolution to approximately 250 nm. This has been accomplished by multiple microscopy developments, including structured illumination and stimulated emission depletion (STED), among others^4^. Although these systems facilitate molecular characterizations at the nanoscale, most pathology departments rely on the resolution of electron microscopy (EM) as super-resolution technologies are considered less cost-efficient and in some cases, cumbersome. For this reason, additional efforts have been made to find alternative approaches that are reliable, reproducible, inexpensive and can provide sufficient resolution for molecular ultrastructural tissue profiling.

One technology that fits these criteria is expansion microscopy (ExM)^1^. Conventional protocols for ExM achieve approximately 4-fold unidimensional isotropic expansion^5^, allowing a resolution of up to 70 nm in tissues^6^ using diffraction-limited microscopy, which can range from laser-based (confocal) to light-emitting diode (LED)-based (widefield (WF)) systems. Interestingly, in this rapidly evolving field, where protocol modifications, for example, iterative ExM^7^, have reached up to 20-fold expansion with 25 nm resolution in vitro and in experimental samples, most studies continue to use 4-fold expansion protocols. It has been proposed^8^ that the main drawbacks of expansion methods over 4-fold are: (1) reductions in the field of view, thereby losing one of the main advantages of ExM over EM; (2) more complex procedures require more quality controls to exclude potential artefacts; (3) reductions in fluorophore intensity; and (4) the need of laser-based systems for optimal performance. For these reasons, we postulate that 4-fold ExM can serve as the foundation of a new approach, which may synergize with a complementary technique to achieve a resolution range compatible with routine clinical diagnostics without the limitations linked to higher expansion ranges.

To bridge this gap, computational image enhancement has emerged as a powerful tool. For example, super-resolution radial fluctuations^2^ (SRRF) provide an open-source method of augmented optical resolution compatible with LED-based systems. Briefly, SRRF requires the acquisition of a time series that will undergo a stepwise process based on the measurement of local radial symmetries and temporal fluctuations over time, resulting in a single image with enhanced resolution in the range of 60–150 nm. To date, SRRF has been primarily used in the field of experimental cell biology^9,10^, as it provides a resolution range that does not offer substantial advantages for clinical pathology compared with EM. Thus, we hypothesize that a combination of ExM and SRRF can provide sufficient resolution for histopathological applications using LED-based WF systems that are more accessible to clinical pathology units and the broad scientific community.

**Fig. 1:**
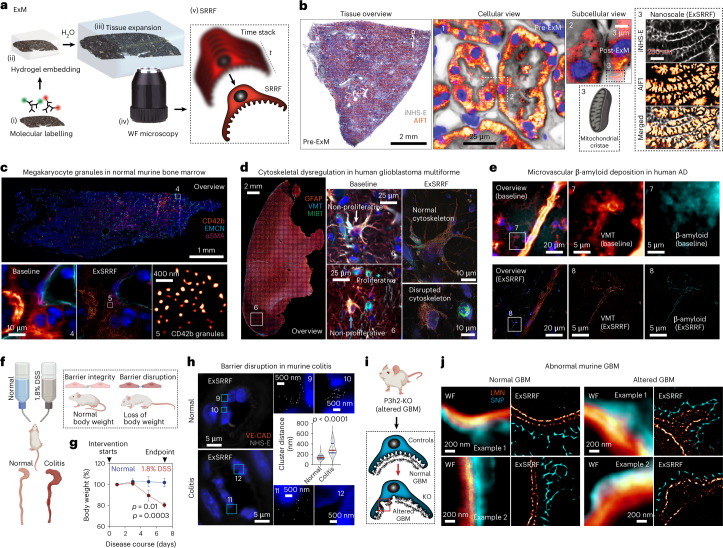
Concept and broad applicability of ExSRRF to clinical and experimental tissues. **a**, Schematic of the ExSRRF workflow, including (i) fluorescent molecular labelling, (ii) hydrogel embedding, (iii) tissue expansion, (iv) LED-based WF microscopy with time-stacked acquisition of the Region of Interest (ROI) and (v) computational image processing using SRRF. **b**, A kidney sample was labelled with pan-protein staining (inverted NHS-ester (iNHS-E)) in combination with a mitochondrial marker (apoptosis-inducing factor 1 (AIF1)). Whole-tissue scanning using LED-based WF systems generates a sample overview with spatial context and molecular information at a cellular level. ExSRRF allows the subcellular identification of hallmark mitochondrial features. **c**, ExSRRF resolves cytoplasmatic CD42b^+^ granules in perisinusoidal murine bone marrow, using endomucin (EMCN) and alpha-smooth muscle actin (aSMA). **d**, ExSRRF reveals cell heterogeneity in human glioblastoma. Active cell proliferation was defined using E3 ubiquitin protein ligase (MIB1; green), and astrocyte cytoskeletal filaments were defined using glial fibrillary acidic protein (GFAP; orange). We provide representative images of a non-proliferative astrocyte with normal cytoskeleton (MIB1^−^GFAP^+^; white arrow), a proliferative cell (MIB1^+^GFAP^–^; magenta arrow) and an astrocyte with disrupted cytoskeleton (MIB1^–^GFAP^+^; cyan arrow); the panels on the right provide zoomed-in views of the panels in the centre. **e**, Microvascular β-amyloid deposition in human brain tissue from a patient with AD, using vimentin (VMT) as a vascular reference label. **f**,**g**, Schematic of murine experimental colitis based on the administration of dextran sulfate sodium (DSS) (**f**), resulting in a marked loss of body weight (*N* = 4 mice per group) (**g**); the dots represent mean and the error bars represent standard deviation. **h**, ExSRRF revealed a progressive loss of VE-CAD clusters (cell–cell connections) in the submucosal intestinal vasculature during colitis. In the violin plots, the red lines represent medians, and the blue lines represent interquartile ranges. **i**, Schematic of a murine model of disrupted glomerular basement membrane (GBM) through P3h2 knockout (KO). **j**, GBM is labelled with laminin (LMN), and the podocyte FPs are labelled with synaptopodin (SNP). ExSRRF-resolved well-organized bilayered LMN distribution is shown under normal conditions, as well as irregular GBM protrusions and focal loss of LMN in KO mice (*N* = 3 mice per group). The ‘baseline’ refers to WF images acquired before expansion and without SRRF enhancement. The statistics were performed using two-tailed unpaired *t*-tests with Welch’s correction.

**Extended Data Fig. 1:**
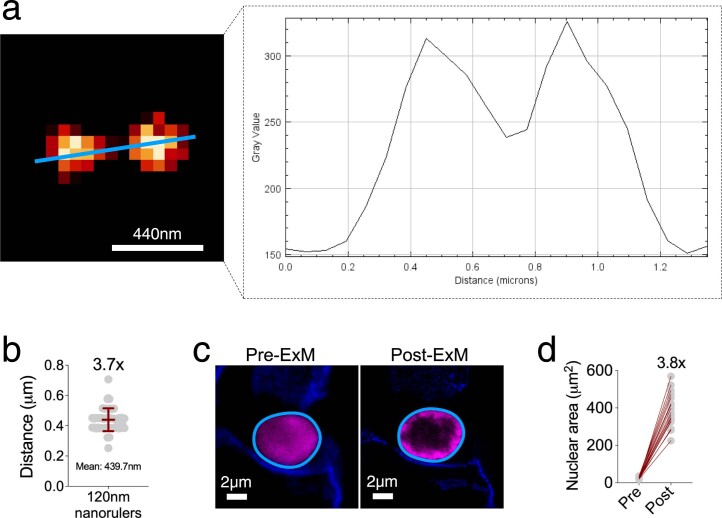
Quantification of expansion factor. (a) Using the 120 nm nanorulers, we measured the distance between intensity peaks after expansion. (b) The mean distance (and SD) between 120 nm nanorulers was calculated, providing an average expansion factor of 3.7x (N = 50). The data is reported as mean ± SD. Then, we used nuclear stainings in tissues (c), the cross-sectional area of individual nuclei was calculated before and after expansion (d), providing an average expansion factor of 3.8x (N = 18).

**Extended Data Fig. 2:**
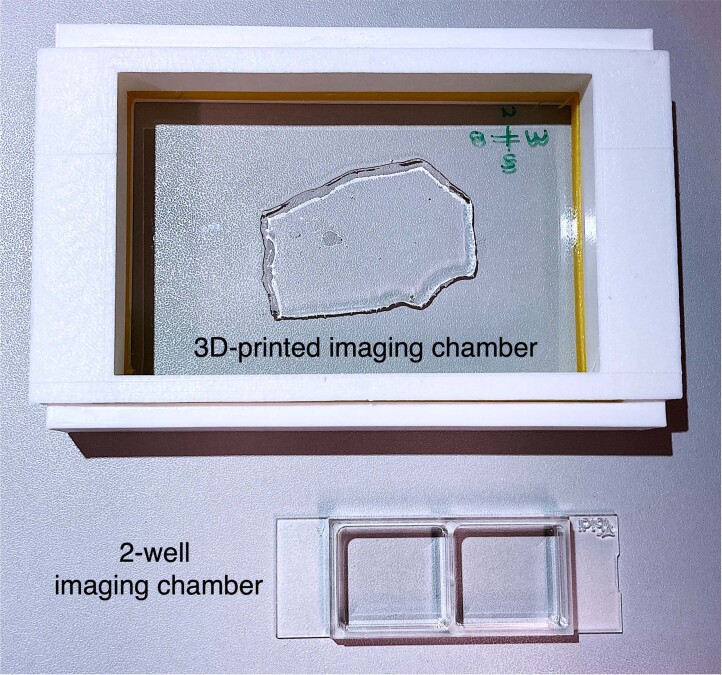
Tissue mounting. Exemplary images of a customized 3D-printed imaging chamber for large samples (top), and a commercial 2-well imaging chamber for small specimens (bottom).

**Extended Data Fig. 3:**
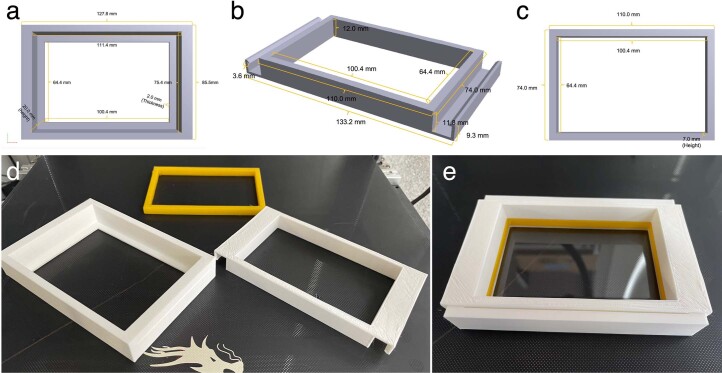
3D-printed imaging chamber. Here, we provide the details of the design of the three components, including (a) frame, (b) lid, and (c) elastic cushion. (d) All 3D-printed parts are shown. (e) The frame is then fitted with a NexterionTM coverslip (see methodology), elastic cushion, and lid.

Here, we introduce expansion-enhanced super-resolution radial fluctuations (ExSRRF; Fig. 1a), a workflow based on molecular fluorescent labelling of tissues that are embedded in a hydrogel, which allows hydration-based expansion (estimated expansion factor range, 3.7–3.8-fold; Extended Data Fig. 1). As previously suggested, we chose this expansion range to preserve a large field of view, and all the additional benefits of 4-fold ExM, including simplicity, reproducibility, mitigation of artefact generation and preservation of fluorescence intensity. After expansion and mounting in either commercial or customized three-dimensional (3D)-printed imaging chambers, depending on the experimental requirements and sample size (Extended Data Fig. 2; additional details are provided in Extended Data Fig. 3 and Methods), LED-based WF microscopy is used to acquire time stacks that are subsequently processed using the SRRF algorithm^2^.

First, ExSRRF was used to analyse archival formalin-fixed paraffin-embedded (FFPE) human tissues as FFPE represents the main preservation and storage condition in clinical and experimental histopathology. Sections were stained with a pan-protein label to obtain EM-like images^11^. However, unlike EM, which is limited by a narrow field of view, predisposing the analysis to sampling bias^12^, a key feature of ExSRRF is the capacity to easily cross biological scales—from entire tissue overviews (centimetres/millimetres) to subcellular compartments (nanometres) without the need of performing additional steps as needed for correlative microscopy. ExSRRF successfully resolved mitochondrial cristae, defined by their morphology and molecular co-labelling (Fig. 1b), within the tubular compartment of the human kidney. Here, we provide additional examples.

Platelets play important roles in systemic haemostasis and inflammation^13^. Megakaryocytes are precursor cells that reside in the bone marrow and form platelets through cytoplasmatic shedding into sinusoidal lumens^14^. ExSRRF resolved CD42b-containing granules from megakaryocytes within the murine bone marrow (Fig. 1c), which is compatible with previous observations^15^. Similarly, the study of heterogeneous tumours with scattered cell infiltration represents an interesting challenge. Glioblastoma is the most aggressive form of glioma with poor prognosis and very limited therapeutic options^16^. Microscopically, it presents regions of necrosis, pleomorphic cells and microvascular proliferation; genetically, it is linked to various deletions, amplifications and point mutations, all of which lead to the activation of multiple critical signalling pathways (for example, disruption of cell-cycle arrest)^17^. ExSRRF identified cellular proliferation and filament dysregulation in human glioblastoma (Fig. 1d), linking morphological changes to cell-cycle profiles.

Human Alzheimer’s disease (AD), a common and highly debilitating neurodegenerative disorder leading to dementia, is associated with the deposition of β-amyloid-containing extracellular plaques^18^, including vascular β-amyloid deposits^19^, which appear to have a distinct pathophysiological pattern compared with extracellular plaque deposition^20^. ExSRRF revealed microvascular β-amyloid deposition, which was not detectable using WF microscopy (Fig. 1e), highlighting the notable increases in signal-to-noise ratio obtained by the attenuation of neighbouring autofluorescence. Similarly, placental villi also represent a challenge for subcellular mapping as they cover a large area, connecting maternal and foetal vascular beds^21^. Von Willebrand factor (vWF), a large multimeric plasma protein that is produced in endothelial cells, plays a central role in haemostasis and inflammation^22^. Long vWF multimers are stored in specialized lysosome-related secretory granules known as Weibel–Palade bodies^23^, which can be clearly visualized using ExSRRF (Supplementary Fig. 1a).

Next, we tested ExSRRF on two well-characterized experimental models of disease. First, the intestinal barrier prevents the luminal contents from crossing into the blood stream^24^. Endothelial junctions (for example, VE-cadherin (VE-CAD)), which are injured in inflammatory colitis^25^, represent a final barrier before systemic circulation. Dextran sulfate sodium (DSS) administration serves as an experimental model of colitis (Fig. 1f), as it exerts direct toxicity to the colonic epithelium^26^, leading to significant weight loss between days 5 and 7, correlating with disease severity (Fig. 1g). ExSRRF identified highly organized individual VE-CAD clusters under normal conditions. In colitis, the distance between individual VE-CAD clusters significantly increased, leading to focal loss and suggesting progressive disorganization (Fig. 1h). Similarly, the kidney filtration barrier can be altered in a genetic mouse model of thin glomerular basement membrane (GBM) disease,^27^ leading to ultrastructural changes in the GBM (Fig. 1i), highlighting the complex interactions between epithelial cells and their surrounding microenvironment. ExSRRF successfully resolved well-organized bilayered laminin distribution within the normal GBM and identified irregular GBM protrusions and focal loss of laminin under pathological conditions (Fig. 1j). To increase the complexity and perform more in-depth analyses of cell–cell and cell–matrix interactions, we propose that adding signalling layers may be advantageous, for example, a combination of in-situ hybridization and antibody-based fluorescence microscopy (Supplementary Fig. 1b), which allowed the visualization of mRNA within protein-based endoplasmic reticulum (ER) networks in murine podocytes.

**Fig. 2:**
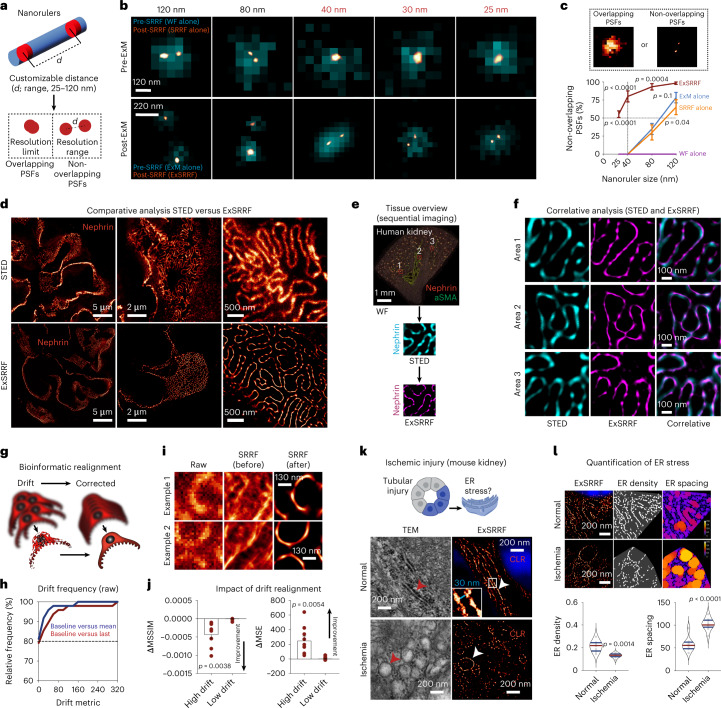
Multilayered validation of ExSRRF. **a**, Nanorulers are equipped with two fluorescently labelled positions at customizable distances (from 25 to 120 nm for this experiment). The resolution range is defined as the smallest distance by which the two spots are resolved without displaying overlapping PSFs. **b**, Comparison among WF, SRRF, ExM and ExSRRF using nanorulers (*N* = 3 replicates per nanoruler). **c**, Quantitative analysis of the percentage of non-overlapping PSFs (*N* = 3 replicates per nanoruler). Data are reported as mean ± standard error of the mean. **d**, STED confirmed ExSRRF outputs using a comparative view of the kidney SD labelled with nephrin. **e**,**f**, Correlative microscopy using STED and ExSRRF in the same sample (stained with nephrin and aSMA) revealed identical structures within the same regions. **g**, Lateral movement (drift) during time-stack acquisition resulted in artefact generation. **h**, Relative frequency of drifts after ExSRRF (*N* = 48). **i**, Performance of SRRF was directly affected by drift artefacts, which were reversed after realignment. **j**, Impact of drift correction measured by deltas in the mean structural similarity index measure (ΔMSSIM) and mean squared error (ΔMSE). Data are reported as mean ± standard error of the mean. **k**, In a murine model of renal IRI, ExSRRF revealed tight ER in control mice (*N* = 7) using calreticulin (CLR) as a marker of the ER lumen; the enlarged inset shows a 30 nm distance between the luminal walls. IRI (*N* = 7) led to dilated ER, which was confirmed by transmission electron microscopy (TEM). **l**, Automated image analysis (image base: *N* = 8 for controls and *N* = 9 for IRI) identified significant reductions in ER density and increases in ER spacing under ischaemic conditions; here the colour coding reflects the local spacing at a pixel level, with 0 being the shortest distance (blue) and 255 being the maximum distance (orange). The violin plots represent the median and quartiles of each distribution. In **c**, we used one-way ANOVA with Holm–Sidak correction for multiple comparisons. In the other panels, the statistics were performed using two-tailed unpaired *t*-tests with Welch’s correction.

A systematic approach was used to characterize the resolution, reliability and performance of ExSRRF. First, we aimed to accurately define the resolution limits of ExSRRF using nanorulers^28^, which are synthetic molecules consisting of fluorescent dyes inserted at predefined distances into DNA origami structures that can be crosslinked and expanded within acrylamide-based hydrogels^29^. The target resolution is achieved when non-overlapping point spread functions (PSFs) can be unambiguously identified (Fig. 2a). In subsequent steps, nanorulers created with defined sizes between 25 and 120 nm were imaged pre- and post-expansion and using different objectives (Supplementary Fig. 2). WF microscopy alone failed to resolve at 120 nm. Furthermore, both ExM and SRRF successfully resolved on their own at 80–120 nm but failed to resolve at 25–40 nm (Supplementary Fig. 3a). Even a laser-based system alone failed to resolve at 120 nm, and ExM combined with laser-based imaging resolved at 120 and 80 nm but failed at 40 nm (Supplementary Fig. 3b). In contrast, ExSRRF successfully resolved nanorulers between 25 and 40 nm (Fig. 2b). The quantification of non-overlapping PSFs revealed that ExSRRF was the only method that showed an average recall of 82% across our entire range of nanorulers, whereas ExM or SRRF alone not only started at lower levels (78% and 65%, respectively, versus 98% for ExSRRF) but showed immediate declines that reached 0% at 40 nm compared with ExSRRF that maintained 80% at 40 nm and remained above 50% at 25 nm (Fig. 2c). Our data suggest that ExSRRF achieves a resolution range of classical super-resolution systems and surpasses its individual components (ExM and SRRF).

**Extended Data Fig. 4:**
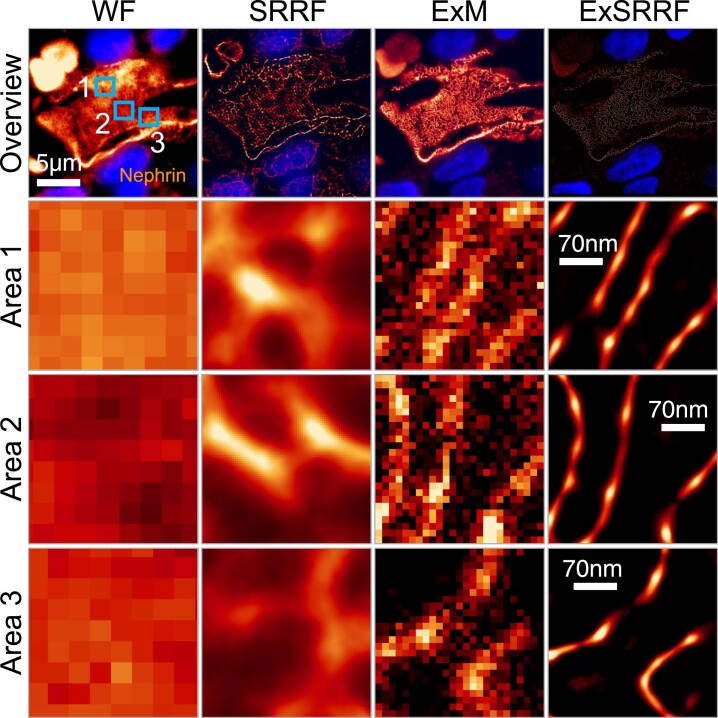
ExSRRF achieves better resolution than its individual components in tissue. The image sequence shows a comparison between the individual components of ExSRRF, namely expansion microscopy (ExM) and SRRF, and widefield (WF) as a negative control and ExSRRF in a known structure (e.g., kidney slit diaphragm labeled with Nephrin).

Next, we used a well-known structure in the mammalian kidney, the slit diaphragm (SD), which has a width of approximately 40 nm^30^, providing a unique opportunity to test ExSRRF in tissues. First, a comparative validation of pre-expansion STED and ExSRRF revealed similar visual patterns of nephrin, a key component of the SD (Fig. 2d). Then, correlative microscopy of STED and ExSRRF was performed, where an overview of the tissue was generated to identify the same areas with pre-expansion STED and subsequently with ExSRRF (Fig. 2e). This approach confirmed that the images obtained with ExSRRF represent biological structures that look identical to those obtained with STED (Fig. 2f). In addition, a direct comparison among WF, ExM, SRRF and ExSRRF within the same regions showcased that ExSRRF also outperforms its individual components in tissues (Extended Data Fig. 4).

**Extended Data Fig. 5:**
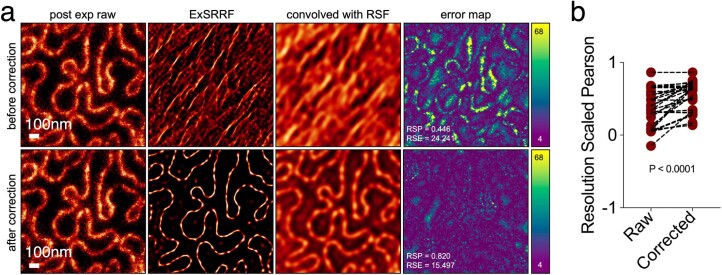
Adjusting potential drift artefacts during image acquisition. (a) The sequence shows images before and after drift correction and the impact this has on image quality and artefact generation using NanoJ-SQUIRREL. RSF: Resolution Scaling Function (b) Quantitative definition of the impact of image drift correction on one of the main NanoJ-SQUIRREL outputs (Resolution Scaled Pearson coefficient or RSP). RSE: Relative scaling error. Statistical testing was performed using a two-tailed paired *t*-test.

One possible drawback of ExSRRF is the potential generation of motion artefacts during time-stack acquisition. For this reason, we propose to realign the image stack using the first image as a reference, thereby mitigating the potential drifts during image acquisition (Fig. 2g). First, we established relative frequencies of drift metrics, which revealed that approximately 80% of the images did not show significant drift-related artefacts (Fig. 2h). The impact of image realignment was determined both visually (Fig. 2i), where the application of SRRF to the raw data with significant misalignments provides clear artefacts, as well as quantitatively using the structural similarity index measure and mean squared error (Fig. 2j). In addition, NanoJ-SQUIRREL^31^ was used as quality control to account for the generation of general artefacts. After drift correction, we observed a significant improvement in the resolution-scaled Pearson coefficient (Extended Data Fig. 5).

**Extended Data Fig. 6:**
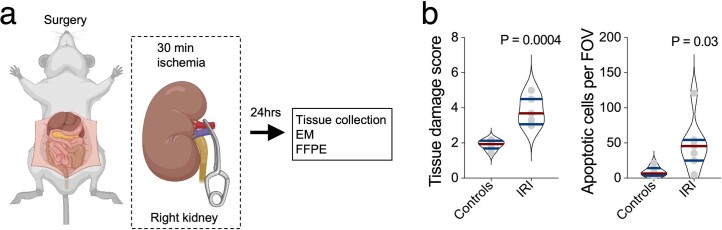
Mouse model of ischaemia reperfusion injury (IRI). (a) Schematic representation of the experimental design. (b) Quantification of tissue damage and apoptosis (N = 7 per group). EM: electron microscopy; FFPE: formalin-fixed paraffin-embedded; FOV: field of view. Violin plots in this figure represent the median and quartiles of each distribution. Statistics were performed using two-tailed unpaired *t*-tests with Welch’s correction.

**Extended Data Fig. 7:**
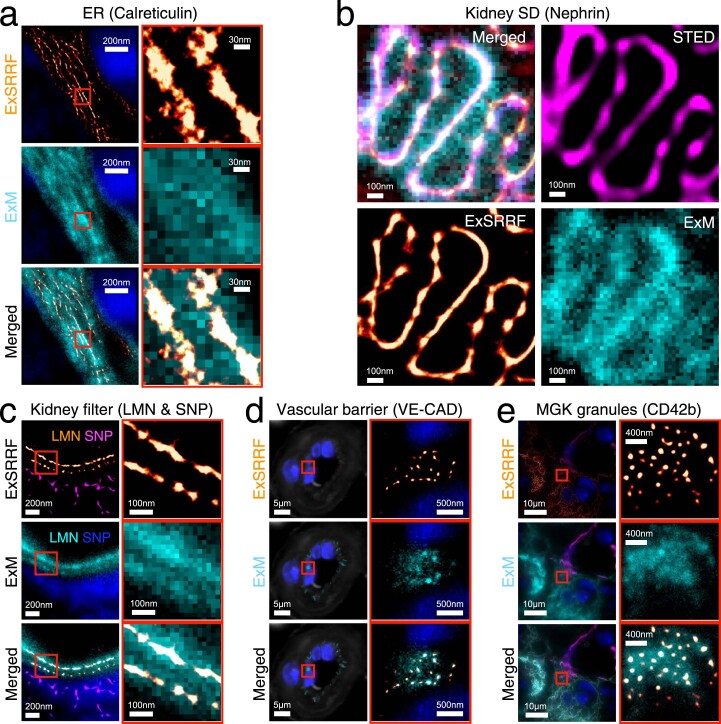
ExSRRF allows visualization of structures that cannot be identified using ExM alone. (a) While ExSRRF resolves murine endoplasmic reticulum (ER) in kidney tubular cells, ExM does not provide sufficient resolution for unambiguous identification. (b) Human kidney slit diaphragm (SD) is fully resolved by STED and ExSRRF but not by ExM alone. (c) ExSRRF shows clear separation of bi-layered basement membrane (Laminin; LMN) within the murine kidney filter (podocyte foot processes labelled with Synaptopodin; SNP), but ExM does not resolve a double layered basement membrane or individual podocyte foot processes. (d) VE-cadherin (VE-CAD) clusters are fully separated by ExSRRF but not by ExM. (e) ExSRRF resolves individual intracellular CD42b^+^ granulae in murine perisinusoidal bone marrow megakaryocytes (MGK), and ExM fails to identify granulae.

**Extended Data Fig. 8:**
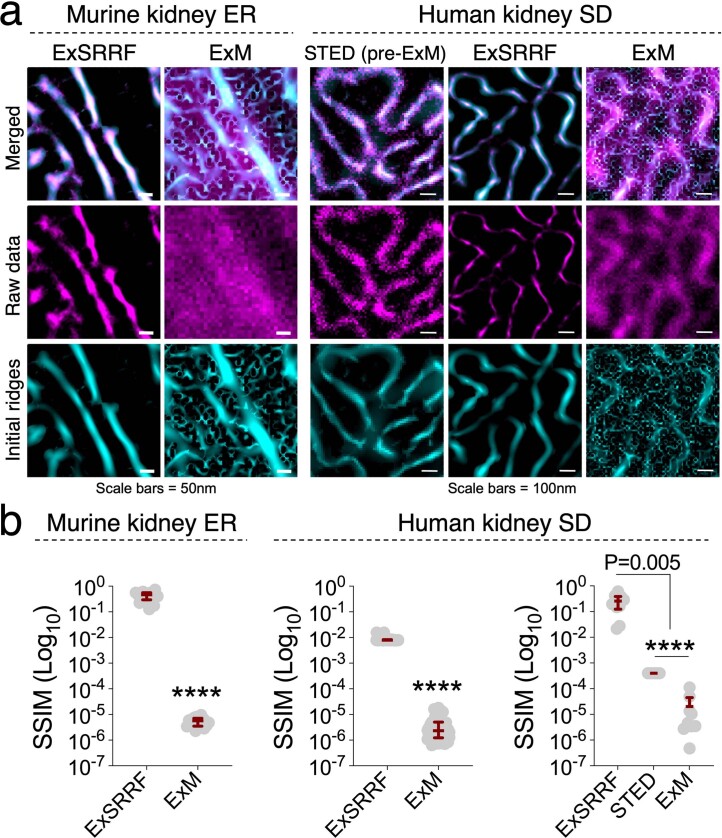
ExSRRF facilitates better segmentation compared to ExM alone and STED. We provide both visual (a) and quantitative (b) confirmation of the resolution gain provided by ExSRRF in comparison to ExM alone. SSIM: structural similarity index measure, ER: Endoplasmic reticulum (N = 17), SD: slit diaphragm (ExSRRF vs ExM: N = 52; and ExSRRF vs ExM vs STED: N = 10), **** represents P < 0.0001. Every grey dot represents one image. Violin plots represent the median and quartiles of each distribution. Statistics were performed using two-tailed unpaired *t*-tests with Welch’s correction when comparing two groups, and a Welch ANOVA with a Dunnett’s multiple comparisons test.

Next, we explored the application of ExSRRF as a visual and quantitative tool in a mouse model of renal ischaemia–reperfusion injury (IRI)^32^, a type of injury that is also highly relevant in multiple other fields, including cardiology and neurology-related vascular biology^33,34^. The ER is a plate- or net-like membrane system with a luminal diameter ranging between 20 and 60 nm^35^ that fulfils essential cellular functions, and displays stress-mediated dilation in IRI^36^. Given its normal luminal diameter, the study of ER requires a resolution range beyond conventional ExM and SRRF. After confirming that our model of IRI induced significant kidney injury (Extended Data Fig. 6), we examined images acquired with ExSRRF using calreticulin, a chaperone protein marking the ER lumen, which mirrored morphological ER features identified by EM (Fig. 2k) and previous reports with luminal space between lamellae of approximately 30 nm. To strengthen the importance of the augmented resolution range of ExSRRF, we also performed an automatic quantitative analysis that identified reduced ER density and increased ER spacing (Fig. 2l) in association with IRI. This experiment also allowed us to highlight the resolution gain provided by ExSRRF compared with ExM alone (Extended Data Fig. 7), which showed significantly lower structural similarity index measures compared with ExSRRF and pre-expansion STED (Extended Data Fig. 8).

**Fig. 3:**
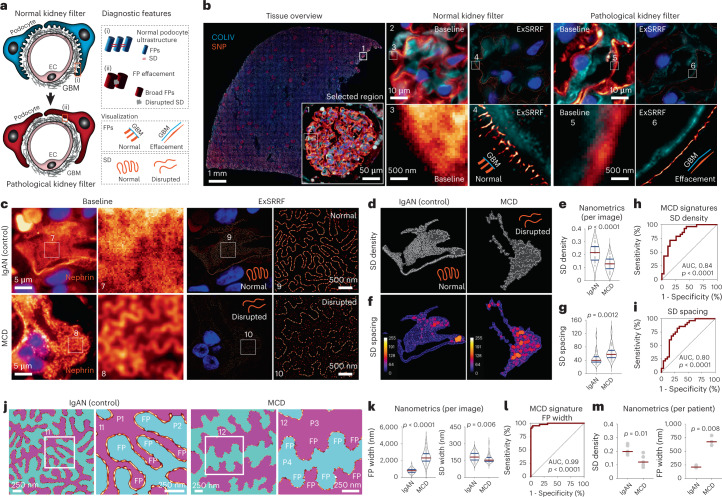
Clinical application of ExSRRF in human kidney biopsies. **a**, Schematic of the kidney filter in health and disease. Cytoplasmatic projections (FPs) of podocytes and their GBM cover the surface of endothelial cells (ECs). FPs interdigitate and interconnect with their neighbours via the SD. On injury, the broadening of FPs (FP effacement) and therefore the disruption of the corresponding SD results in a leaky kidney filter with corresponding diagnostic features. **b**, ExSRRF identified both normal and effaced FPs using synaptopodin (SNP) (labelling FPs; orange) and collagen IV (COLIV) (labelling the GBM; cyan). **c**, ExSRRF resolved the SD (orange) in patients with MCD compared with control patients with immunoglobulin A nephropathy (IgAN) using nephrin. **d**–**g**, Automated image analysis provided nanoscale morphometrics (nanometrics), including SD density (*N* = 27 for IgAN and *N* = 28 for MCD for **d** and **e**) and spacing (*N* = 27 for IgAN and *N* = 28 for MCD for **f** and **g**); here the colour coding reflects the local spacing at a pixel level, with 0 being the shortest distance (blue) and 255 being the maximum distance (orange). **h**,**i**, Signature of MCD was identified using SD density (**h**) and spacing (**i**) per image. **j**, ExSRRF outputs were used to reproduce the perspective obtained using scanning electron micrographs; adjacent podocytes (P1 and P2 in IgAN; P3 and P4 in MCD) were pseudo-coloured with cyan or magenta to reveal the interdigitation pattern between neighbouring FPs. **k**, FP (*N* = 54 for IgAN and *N* = 42 for MCD) and SD (*N* = 52 for IgAN and *N* = 41 for MCD) widths were calculated per image. **l**, Signature of MCD was identified using the FP width per image. **m**, Average nanometrics per patient confirm our results (SD density, *N* = 5 per group; FP width, *N* = 3 per group). The red lines represent the mean values per condition. In this figure, "baseline" refers to WF images acquired before expansion and without SRRF. The violin plots represent the median and quartiles of each distribution. Comparisons of two groups were performed using two-tailed unpaired *t*-tests with Welch’s correction. The receiver operating characteristic curves were generated, the corresponding standard errors were calculated, and *p* values were determined from the normal distribution (two-tailed).

**Extended Data Fig. 9:**
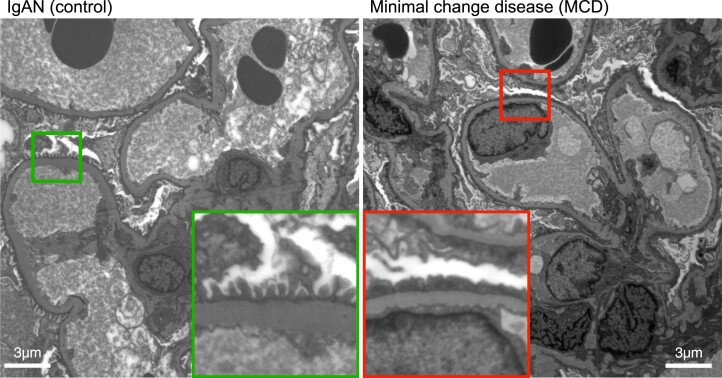
Electron microscopy images used in clinical diagnosis of minimal change disease. Overview and enlarged panels show areas of normal foot process morphology (green) and foot process effacement (red). FP(s): foot process(es); GBM: glomerular basement membrane.

**Extended Data Fig. 10:**
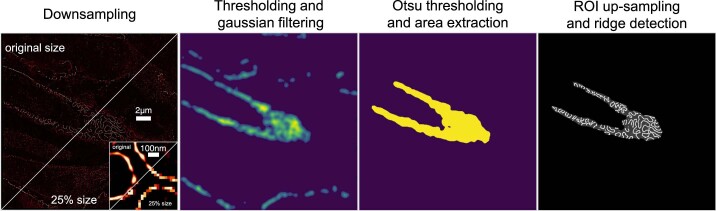
Automatic segmentation after ExSRRF in human kidney biopsies. This sequence describes the image processing steps necessary for automatic segmentation of the kidney slit diaphragm in human kidney biopsies.

Biopsy specimens represent a pillar of the diagnostic process in clinical pathology. For example, subcellular changes in kidney biopsies provide diagnostic features that define disease states and guide therapy. Podocytes are essential components of the glomerular filter. When injured, they retract their cytoplasmic projections (foot processes (FPs)), which broaden and fuse in a pathological change known as FP effacement^37^. In addition, FPs are interconnected by the SD^38^ that is directly disrupted during the effacement process (Fig. 3a). Previous work has identified detailed spatial information of the podocyte proteome^39,40^, providing specific tools for the molecular labelling of both FPs and the SD. We used a combination of collagen type IV (labelling GBM), and synaptopodin (labelling FPs) to compare an EM-defined normal podocyte ultrastructure (controls) with those from patients with EM-defined podocyte alterations (minimal change disease (MCD); Extended Data Fig. 9). ExSRRF was able to resolve normal FPs that have a width of approximately 200 nm, but are separated from each other by the SD (approximately 40 nm)^38^. Importantly, ExSRRF detected the effacement of FPs in pathological samples (Fig. 3b), a key diagnostic feature provided in EM reports. Then, we confirmed marked alterations in the SD of patients with MCD (Fig. 3c), which were not visible in controls and were not detectable via WF microscopy. Importantly, the SD cannot be appropriately evaluated by routine transmission EM, giving ExSRRF an additional advantage. Next, we developed an algorithm for the automatic segmentation of SD (Extended Data Fig. 10), which uncovered differences in the SD density (Fig. 3d,e) and dilation (Fig. 3f,g). This morphometric analysis performed at the nanoscale, which we now call ‘nanometrics’, was used to define a disease signature per image (Fig. 3h,i). ExSRRF data were used to reproduce the perspective obtained using scanning electron micrographs—adjacent podocytes were pseudo-coloured to visualize the normal interdigitation pattern of FPs in controls, and FP broadening in MCD (Fig. 3j). The SD was clearly observed between adjacent FPs in controls, but it was disrupted in the MCD. Furthermore, the width of FPs and SD revealed a pattern of FP broadening and disruption of the SD in MCD (Fig. 3k). The FP width was also used to identify a disease signature (Fig. 3l), which was the best performer among all the nanometrics (area under the curve, 0.99; *p* < 0.0001). Finally, we generated the average nanometrics per patient, which revealed significant differences between the controls and MCD (Fig. 3m). Together, our data show that ExSRRF can facilitate the identification and quantification of diagnostic features in podocyte injury for human kidney biopsies, providing a workflow for the assessment of kidney diseases that can serve as an example applicable to any other field in pathology, especially in conditions where EM-like resolution is needed.

In summary, ExSRRF is a flexible, scalable, inexpensive and robust method for the multilayered profiling of experimental and clinical specimens across large biological scales. We propose that ExSRRF has the potential to expand the boundaries of experimental histopathology at nanoscale and to integrate super-resolution microscopy and clinical pathology, providing unprecedented subcellular molecular, morphological and quantitative insights, thereby paving the way for the development of molecular nanopathology and standardized nanometrics to better diagnose, stratify and treat patients.

## Methods

### Human samples

FFPE tissues were collected according to institutional protocols. The control kidney specimens were collected in collaboration with the Division of Nephrology and Clinical Immunology, RWTH Aachen University. Kidney biopsies from patients with MCD and IgAN were obtained from the Hamburg Glomerulonephritis Registry (https://www.sfb1192.de/en/register). Ethical approvals were obtained from the Institutional Review Board of the RWTH Aachen University Medical Center (EK-016/17); the Ethik-Kommission der Ärztekammer Hamburg; and the local ethics committee of the Chamber of Physicians in Hamburg (PV4806). All of them are in accordance with the ethical principles stated by the Declaration of Helsinki.

In addition, specimens from patients with glioblastoma and AD were provided by the Institute of Neuropathology, University Medical Center Hamburg-Eppendorf. The study was reviewed by the ethics committee of the Hamburg Chamber of Physicians (WF72/17). A sample from human placenta was provided by the Division of Experimental Feto-Maternal Medicine, Department of Obstetrics and Fetal Medicine, University Medical Center Hamburg-Eppendorf, and approved by the Hamburg Chamber of Physicians (PV7312).

### Murine samples

FFPE murine normal bone marrow was provided by the Department of Developmental Biology and Oncode Institute at Erasmus Medical Center Cancer Institute. Tissue collections were approved by the Animal Welfare/Ethics Committee of the EDC in accordance with legislation in the Netherlands (AVD1010020173387).

Control murine kidney tissue and experimental IRI tissue were collected in the Department of Medicine, University Medical Center Hamburg-Eppendorf. All the procedures were performed under the approval of the University Hospital Hamburg-Eppendorf and the Hamburg State Department for Animal Welfare under application no. N002/2020. Mouse anaesthesia: 30 min before surgery, 0.05 mg kg^–1^ buprenorphine was subcutaneously injected. At the time of surgery, narcosis was induced and maintained using isoflurane (5% isoflurane in pure O_2_ for induction, 2% isoflurane in O_2_ for maintenance). Induction of IRI: mice were anaesthetized and placed onto a heating pad (39 °C). The abdomen was opened via a median laparotomy and the renal artery of the right kidney was clamped for 30 min (micro-serrefine clamp, Fine Science Tools; 18055-03). The abdomen was filled with 0.9% NaCl and temporarily closed using Parafilm (Bemis). After this time, the clamp was removed, the abdomen was closed using a 3.0 suture and anaesthesia was stopped. Animals were placed back into their respective cages and had free access to soft chow and water, sweetened (eight drops of Natreen Classic) with added metamizole (1.3 mg ml^–1^, Ratiopharm). Endpoint: after 24 h, anaesthesia was induced again, and the abdomen was opened. Abdominal vessels were exposed, and the kidney perfusion was fixed via the abdominal aorta using 4% paraformaldehyde in 1× phosphate-buffered saline (PBS) at 200 mmHg for 5 min. The kidneys were harvested and stored in 4% PFA at 4 °C until further analysis.

Murine colon was obtained from controls and an experimental model of colitis. All the procedures were performed under the approval of the University Hospital Hamburg-Eppendorf and the Hamburg State Department for Animal Welfare under application no. TVA17/13. Mice were housed under specific pathogen-free conditions in individually ventilated cages with standard food and water *ad libitum*. Age- and sex-matched B6 wild-type mice between 8 and 12 weeks were used. During experiments, the mice were monitored daily. Intervention group mice were fed 1.8% DSS salt (MP Biomedicals; molecular weight, 36,000–50,000 Da) in drinking water for 7 days to induce colitis, whereas control mice were fed normal water^41^.

Archival tissues from an experimental model of disrupted GBM were obtained from a previous experiment. The generation of this mouse line was described in the original publication, together with ethical approval by Regierungspräsidium Freiburg (G16-122) and BGV Hansestadt Hamburg (Ü 004/2018).

### De-waxing and antigen retrieval

Paraffin sections were cut at a thickness of 2–4 µm and mounted on Superfrost Plus slides. Then, all the samples were sequentially immersed in xylene 3× (10 min each) followed by an ethanol series (5 min each) of 3× 100%, 2× 70%, 1× 50% and finally 3× (5 min each) in double-deionized water. Antigen retrieval was performed with Agilent DAKO Target Retrieval Solution at pH 9 (catalogue no. S236884-2) in a Braun Multiquick FS20 steamer for 15 min, followed by cooling down to room temperature for 30 min. The sections were then incubated in Agilent wash buffer solution (catalogue no. K800721-2) for 15 min at room temperature.

### Fluorescent immunolabelling

Samples were incubated with primary antibodies at concentrations according to vendor’s guidelines in Agilent antibody diluent solution (catalogue no. K800621-2) overnight at 4 °C, followed by three times washing for 5 min with Agilent wash buffer solution. Then, sections were incubated with appropriate secondary antibodies as well as directly conjugated primary antibodies at concentrations according to vendor’s guidelines with DAPI (Sigma-Aldrich D9542) at a final concentration of 1 μg ml^–1^ in Agilent antibody diluent solution for 1 h at room temperature and washed again three times for 5 min with Agilent wash buffer solution. Pan-protein staining was performed using NHS-ester (succinimidyl ester) conjugated with Alexa Fluor 555 (Invitrogen A20009) according to vendor’s guidelines. The following secondary antibodies were used: goat anti-guinea pig Alexa Fluor 488 (Invitrogen A11073), goat anti-guinea pig Alexa Fluor 555 (Invitrogen A21435), goat anti-mouse IgG1 Alexa Fluor 488 (Invitrogen A21121), goat anti-rabbit Atto 647N (Sigma-Aldrich MFCD06798562), goat anti-rat Alexa Fluor 555 (Invitrogen A21434) and streptavidin Alexa Fluor 555 conjugate (Invitrogen S21381). After immunostaining, the samples were mounted with ProLong Gold (Invitrogen P36930) for pre-expansion imaging.

### Fluorescent in-situ hybridization

Fluorescent in-situ hybridization was performed in FFPE murine kidney samples using the RNAscope Multiplex Fluorescent Reagent Kit V2 Assay (catalogue no. 323100) according to the manufacturer’s guidelines and as previously described^42^. RNAscope probe NPHS-1 from Advanced Cell Diagnostics (catalogue no. 43357) was used to detect NPHS-1 mRNA and Opal 570 dye from Akoya Biosciences (catalogue no. OP-001003; dilution, 1:1,500) was applied for signal development.

### Coverslip removal

Previously immunolabelled sections that were mounted and imaged were briefly immersed in xylene for 5 min at room temperature, after which the coverslip was carefully removed using a razor blade. The mounting media were washed off with PBS for 5 min at room temperature.

### Primary antibodies

Primary antibodies and their respective species and dilutions used in this study are as follows: AIF1 (Cell Signaling Technology 5318; rabbit, 1:200), aSMA/FITC conjugate (Abcam F3777; mouse, 1:200), calreticulin (Abcam ab92516; rabbit, 1:200), CD42b (Abcam ab227669; rabbit, 1:200), collagen IV (Abcam ab6586; rabbit, 1:200), cytokeratin 8 (R&D Systems MAB3165; mouse IgG1, 1:200), endomucin (Santa Cruz Biotechnology sc-65495; rat IgG2a, 1:200), GFAP (Thermo Fisher Scientific 14-9892-82; mouse IgG1, 1:200), MIB1 (Abcam ab124929; rabbit, 1:200), nephrin (Progen GP-N2; guinea pig, 1:100), synaptopodin (Synaptic Systems 163 004; guinea pig, 1:100), β-amyloid (Thermo Fisher Scientific 715800; rabbit, 1:200), vimentin (Progen GP53; guinea pig, 1:200), vWF (Agilent A008229-2; rabbit, 1:200), laminin (Abcam ab11575; rabbit, 1:200) and CD144 VE-CAD (Thermo Fisher Scientific 14-1441-82; rat, 1:200).

### Tissue expansion

Protocols for tissue expansion for enhanced optical resolution in thin sections have been previously described^6^. Briefly, immunolabelled and fluorescence in-situ hybridized sections underwent anchoring treatment with 0.1 mg ml^–1^ Acryloyl-X, 6-((acryloyl)amino)hexanoic acid, succinimidyl ester (Invitrogen A20770). On the other hand, no further anchoring treatment was required for fluorescence in-situ hybridized sections as the RNAscope Multiplex Fluorescent Reagent Kit V2 Assay utilizes Akoya Biosciences proprietary Tyramide signal amplification technology, resulting in the covalent deposition of OPAL dye molecules to nearby proteins. Acryloyl-X aliquots were dissolved in anhydrous DMSO at a concentration of 10.0 mg ml^–1^ for long-term storage at −20 °C and diluted in PBS to the final concentration of 0.1 mg ml^–1^ at room temperature for 12 h. Tissue sections were then embedded into a gelling solution consisting of 1× PBS, 2 M NaCl, 8.625% sodium acrylate (Sigma-Aldrich 408220), 2.500% acrylamide (Sigma-Aldrich A3553), 0.100% *N*-*N*′-methylenbis-(acrylamide) (Sigma-Aldrich 146072), 0.010% 4-hydroxy-2,2,6,6-tetramethyl-piperidin-1-oxyl (Sigma-Aldrich 176141), 0.200% *N*,*N*,*N*′,*N*′-tetramethylethylenediamine (Sigma-Aldrich T9281) and 0.200% ammonium persulfate (PanReac AppliChem, A1142). Embedded sections in the gelling solution were then incubated at 4 °C for 30 min to allow the penetration of gelling solution into the tissue. After that, gelling chambers, each consisting of two coverslips as spacers on either side of the tissue to prevent compression and a third coverslip on top of the tissue, were constructed around the tissue. In addition, sections were incubated in a humidified oven at 37 °C for 2 h to complete gelation. Next, the gelling chambers were removed, and specimens were incubated in 8 U ml^–1^ proteinase K (Sigma-Aldrich P2308) in a Tris/EDTA-based digestion buffer (50 mM Tris (pH 8), 25 mM EDTA, 0.5% Triton X-100 and 0.8 M NaCl) at 60 °C for 4 h. Following digestion, gel-embedded tissue sections were placed in double-deionized water at room temperature for 60 min to allow for isotropic expansion. After completing expansion, the tissues were removed from the double-deionized water and mounted in glass-bottom chamber slides (Ibidi µ-Slide two-well glass bottom; catalogue no. 80287) for subsequent imaging.

### Nanoruler mounting and expansion

ExM-compatible nanorulers, equipped with acrydite groups to allow for covalent binding to the polymer matrix during gelation and biotin anchors to allow for immobilization on a bovine serum albumin (BSA)–biotin/NeutrAvidin surface, were custom ordered from GATTAquant DNA Nanotechnologies. First, to allow for the immobilization of nanorulers, glass slides were coated with BSA–biotin/NeutrAvidin. More precisely, the glass slides were first washed three times with 1,000 μl PBS, after which they were incubated with 200 μl Pierce BSA, biotinylated (Thermo Scientific 29130) at a concentration of 1 mg ml^–1^, dissolved in PBS, followed by washing another three times with 1,000 μl PBS. Tissues were then incubated with 200 μl NeutrAvidin solution (1 mg ml^–1^ NeutrAvidin Protein (Thermo Scientific 31000) dissolved in PBS) for 5 min. The NeutrAvidin solution was washed by applying three times 1,000 μl PBS, supplemented with 10 mM magnesium chloride. ExM-compatible nanoruler stock solutions were diluted in a ratio of 1:10 in PBS, supplemented with 10 mM magnesium chloride and then applied to BSA–biotin/NeutrAvidin-coated glass slides, incubated for 30 min at room temperature, followed by three washing steps with 1,000 μl PBS supplemented with 10 mM magnesium chloride. The slides were mounted in PBS supplemented with 10 mM magnesium chloride for pre-expansion imaging.

After pre-expansion image acquisition, coverslips were removed. In analogy to the tissue expansion protocol described above, the nanorulers were embedded in the ExM gelling solution and incubated at 4 °C for 30 min, followed by the construction of gelling chambers, after which the gelation step was performed by incubation in a humidified oven at 37 °C for 2 h. After completing gelation, gelling chambers were removed and as a digestion step, the nanorulers were denatured to allow for subsequent expansion by incubation in a 50% formamide (Sigma-Aldrich F9037) solution for 2 h at room temperature, followed by incubation at 4 °C for 12 h. The denatured nanorulers were then placed in double-deionized water at room temperature for 60 min to allow for isotropic expansion. After expansion, the samples were removed from the double-deionized water and mounted in glass-bottom chamber slides (Ibidi µ-Slide two-well glass bottom; catalogue no. 80287) for post-expansion imaging.

### Imaging of nanorulers

Pre- and post-expansion LED-based WF imaging of nanorulers was performed using the THUNDER Imager 3D Live Cell and 3D Cell Culture (Leica Microsystems) fitted with a ×100 objective (numerical aperture (NA), 1.47). LED intensity and exposure times for each condition were systematically optimized. To allow for subsequent pre- and post-expansion SRRF processing, time-stacked images (with each time stack consisting of 50 images) were obtained. Pre- and post-expansion confocal imaging of the nanorulers was performed using the Zeiss LSM 800 confocal microscope with AiryScan using the optimized ×63 objective (NA, 1.4) at 12-fold digital zoom with subsequent AiryScan processing using the ZEN2.6 (blue edition) software.

### Assessment of nanoruler PSF separation

To evaluate the separation of the nanoruler PSF after image acquisition, time-stacked imaging files from all the groups (120–25 nm, pre- and post-expansion) were first pseudonymized for further unbiased quantitative analysis by an independent observer without involvement in sample preparation and image acquisition. From these time stacks, 16–20 regions covering one nanoruler were randomly selected per group based on the raw imaging data, after which SRRF processing was performed. Both raw and SRRF-processed images then underwent supervised histogram adjustments to determine nanoruler PSF separation, which was defined as two fluorescent maxima that were clearly separated by at least one pixel without any signal.

### Assessment of expansion factor and reporting

To evaluate the expansion factor, distances between the two fluorescent probes of the nanorulers were measured using the ImageJ2 Version 2.3.0/1.53q plot profile tool. A line positioned centrally through both fluorophores was drawn and the profile of pixel intensity along that line was generated. Thereafter, the distance between the two intensity maxima, depicting the fluorophores’ centres, was calculated. As in a previous report^6^ as well as a thorough validation of the expansion factor at the nanoscale (using nanorulers), for all the ExSRRF images, correlative pre- and post-expansion images of the region of interest (ROI) were obtained to reference post-expansion distances to pre-expansion biological distances, confirming the stability of the expansion factor at a micrometre scale.

### Pre- and post-expansion imaging of tissues

Pre- and post-expansion LED-based WF imaging of tissues was performed using the THUNDER Imager 3D Live Cell and 3D Cell Culture (Leica Microsystems). Low-magnification pre- and post-expansion whole-tissue overviews were performed with a ×20 objective (NA, 0.40). High-magnification pre- and post-expansion images were obtained using multiple objectives, including a ×40 objective (NA, 1.10), ×63 objective (NA, 1.10) and ×100 objective (NA, 1.47) after optimizing the LED intensity and exposure times. To enable post-expansion SRRF processing, time stacks (each consisting between 20 and 200 images depending on the experimental requirements) were obtained for each ROI.

### Time-stack image registration and movement correction

To correct potential movement during imaging, images within each time stack were first registered in Python 3 using scikit-image^43^ (https://scikit-image.org/) and image registration libraries (https://image-registration.readthedocs.io/en/latest/). Each image in the stack was separately aligned to the first image in the time stack using a reference channel containing our main structure of interest. In each image of the reference channel, the structures were first smoothed using a Gaussian filter with a standard deviation of 1. Then, the lower 90 percentile of the pixel values were set to 0, only keeping the upper 10 percentile of pixel values. Subsequently, the histogram of each image in the time stack was adjusted to match the reference histogram. Finally, the shift between the two images was obtained with the function chi^2^ shift from the image registration library, which uses the discrete Fourier transform upsampling method. This shift was finally removed from all the channels of the image, which were cropped and padded to retain the original size and were then reconstituted into an image stack.

### SRRF and NanoJ-SQUIRREL

SRRF processing of raw data was performed using the Fiji imaging software Version 2.3.0/1.53q (Max Planck Institute of Molecular Cell Biology and Genetics) in combination with the NanoJ-SRRF plug-in.

For the pre- and post-expansion nanoruler SRRF processing, raw time-stacked files were processed using the following SRRF settings. Ring radius, 0.5; radiality magnification, 10; axes in ring; 8; temporal analysis; temporal radiality, average radiality; remove positivity constraint ‘disabled’; renormalize ‘disabled’; do gradient smoothing ‘disabled’; weighting, do intensity weighting ‘active’; do gradient weighting ‘disabled’; corrections, minimize SRRF patterning ‘active’; fast linearize SRRF ‘disabled’.

For all other tissue-based images, raw time-stacked files or histogram-adjusted files were processed using the following settings. Ring radius, 0.5–2.0; radiality magnification, 3–10 (depending on the desired magnification); axes in ring, 2–8 (depending on the desired magnification); temporal analysis; temporal radiality, average radiality; remove positivity constraint ‘disabled’; renormalize ‘disabled’; do gradient smoothing ‘active’; weighting, do intensity weighting ‘active’; do gradient weighting ‘disabled’; corrections, minimize SRRF patterning ‘active’; fast linearize SRRF ‘disabled’. For the processing of the inverted NHS-ester channel (Fig. 1b), the NHS-ester signal was first inverted using the ‘Invert’ function in Fiji, after which SRRF processing was performed (as described above). For the assessment of quality improvement by the image registration process, the super-resolution quantitative image rating and reporting of error locations (SQUIRREL) algorithm was used. Corresponding ROIs were selected in both raw and registered datasets. After being processed with the SRRF algorithm, the obtained super-resolution images were compared with the first image of each associated stack using the SQUIRREL algorithm. The generated error maps were used to visually highlight regions of high discrepancy, whereas resolution-scaled Pearson coefficients were utilized for the quantitative comparison between raw and registered datasets.

### Adjusting image orientation for figure preparation

Pre-expansion and post-expansion images (raw and SRRF-processed images) were rotated using Fiji to obtain a similar orientation of the structures in the *X* and *Y* axes. This approach did not generate visual artefacts, although discrete rotation artefacts at the edges of the image in areas without the true signal cannot be excluded in all the cases.

### Quantification of FP and SD width

To measure the SD width in human kidney biopsies stained with nephrin, the ExSRRF images first underwent automated thresholding using the ImageJ threshold tool in combination with the default algorithm and auto-adjusted. The SD width was then measured using the ImageJ plot profile tool. A line positioned at a 90° angle through the thresholded SD was drawn at multiple random positions along the SD followed by a plot profile analysis of the signal width. To measure the FP width in human kidney biopsies, ExSRRF images from nephrin-stained human kidney biopsy samples first underwent stepwise ImageJ-based processing for indirect morphological identification and segmentation of FPs. First, thresholding using the default algorithm in the auto-adjust mode was performed, followed by image inversion, and watershed segmentation, based on which further supervised segmentation was performed resulting in the identification of FPs separated by the SD. The FP width was then measured using the ImageJ plot profile tool by drawing a line at a 90° angle through the FP at multiple random positions followed by a plot profile analysis of the signal width.

### Automated quantification of SD density and dilation

To quantify the SD density, a multistep process was developed in Python 3 using the scikit-image library^43^. First, the ROI was extracted, where the structures of interest are present. Then, the structures of interest were extracted using ridge detection and post-processing. Finally, the density of these structures was calculated within the extracted ROI. The steps and parameters in this process were optimized on two images from the IgAN samples and one image from the MCD samples and only then extended to all the other images. The results were first evaluated visually before checking the quantitative densities.

To extract the ROI, the image was first downsampled to 25% of its original size. The image was then thresholded to remove low-level noise and smoothed using a Gaussian filter with a standard deviation of 8, followed by Otsu thresholding. Mathematical morphology (binary filling of holes and binary closing) was applied next. Finally, all the connected areas were extracted and only those larger than a certain threshold (5,000 pixels) were retained. The resulting ROIs were upsampled again to the original image size.

SD segmentation was performed with the help of ridge detection using the Meijering ridge detector^44^. The resulting ridges were thresholded to only retain values larger than 0.2 and mathematical morphology (opening) was applied. SD densities were finally obtained as the ratio of pixels associated to the SD within the ROI. In addition, SD dilation was calculated in a similar fashion to trabecular thickness and spacing^45^, a commonly used plug-in for bone density analysis. Local spacing at a pixel of the image is the diameter of the greatest circle that fits within the space between ridges, and which is a part of the ROI and contains the point. The implemented ImageJ macro calculated the mean, median, standard deviation and area fraction of ridge spacing. For this study, we used the median per image.

### Automated quantification of ER stress

To quantify the damage caused by IRI, a multistep process similar to the calculation of SD density and dilation was developed in Python 3 using the scikit-image library^43^. First, the ROI was extracted, where the structures of interest are present. Then, the structures of interest were extracted using ridge detection and post-processing. Finally, the density of these structures was calculated within the extracted ROI. The steps and parameters in this process were adapted from the calculation of SD density and dilation and optimized on one image from the control samples and one image from the IRI samples and only then extended to all the other images. The results were first evaluated visually before checking the quantitative densities.

To extract the ROI, the image was first downsampled to 25% of its original size. The image was smoothed using a Gaussian filter with a standard deviation of 8 and a Gaussian filter with a standard deviation of 10, followed by Otsu thresholding. Mathematical morphology (binary filling of holes) was applied next. Finally, all the connected areas were extracted and only those larger than a certain threshold (5,000 pixels) were retained. The resulting ROIs were upsampled again to the original image size.

Segmentation of calreticulin-based ER networks was performed with the help of ridge detection using the Meijering ridge detector^44^. The resulting ridges were thresholded to only retain values larger than 0.25; small objects with a size of less than 125 pixels were removed, and mathematical morphology (opening) was applied. The ER densities were finally obtained as the ratio of pixels associated to the ER within the ROI. In addition, ER dilation was calculated in a similar fashion to trabecular thickness and spacing^45^, a commonly used plug-in for bone density analysis. Local spacing at a pixel of the image is the diameter of the greatest circle that fits within the space between the ridges, and which is a part of the ROI and contains the point. The implemented ImageJ macro calculated the mean, median, standard deviation and area fraction of ridge spacing.

### Evaluation of drift corrections

The registrations of time stacks were evaluated by taking into account the offset by which the frames had to be shifted during the registration process. For each frame in the time stack, the offset distance (2-norm) of the *x* and *y* offsets was calculated using the linear algebra function in the NumPy library (https://numpy.org/) using Python 3. These time-wise offsets were summarized in the mean offset and last offset, referring to the offset of the final frame, usually the largest offset in a time stack. For each time point, the mean structural similarity index measure (MSSIM) and mean squared error (MSE) were calculated using the scikit-image library^43^. These metrics were calculated for both original and registered frames with respect to the first frame (reference frame for the registrations), only taking into account the overlapping regions of the image to allow comparability. They were summarized in the mean and last MSSIMs, and the mean and last MSEs before and after registration for each time stack. The values after registration were subtracted from the ones before registration to measure the impact of registration. The MSSIM is larger for more similar structures, which means that a negative change indicates an improvement through registration, whereas the MSE is smaller for more similar images; thus, a positive change indicates improvement.

A standard deviation (s.d.) in the offset (based on 0.5 × s.d. from the mean) was used as the determining factor to split the time stacks into two groups: low drift (the vast majority) and the ones with more registration needed with offsets larger than 0.5 × s.d. were defined as high drift. The two groups were then compared in terms of the improvement achieved during registration. All the codes were implemented in Python 3.

### Evaluation of resolution quantification for reliable segmentation

To evaluate the sharpness and suitability of the images to be used for further analysis of the structures, the raw ExM, ExSRRF and STED images were compared with the initial ridges detected on each image using the structural similarity index measure. A structural similarity index measure closer to 1 indicated better structural similarity between the images and ridges, thus indicating that there is better correspondence between the computed ridges and structures from the raw images.

### Transmission electron microscopy

Murine kidney tissues were briefly fixed with 4.0% PFA and 2.5% glutaraldehyde in 0.1 M cacodylate buffer (pH 7.4) in situ at room temperature, and then dissected, removed and cut into pieces of 1 mm^3^ and fixed for 48 h in the same solution at 4 °C. The tissue blocks were contrasted using 1% OsO_4_ (Roth 7436.1) at room temperature for 1 h and 1% uranyl acetate (Polysciences 21447-25) in 70% ethanol at room temperature for 1 h. After dehydration, tissue blocks were embedded in epoxy resin (Durcopan ACM, Sigma-Aldrich 44611), and ultrathin sections of 50 nm thickness were cut using a Leica EM UC6 ultramicrotome (Leica Microsystems). The sections were imaged using a Zeiss 910 transmission electron microscope and analysed using ITEM software Version 5.2 (build 4768).

Human kidney biopsies were dissected according to standard operating procedures during diagnostic work up and were transferred from 4% formaldehyde into a cacodylate buffer together with sucrose for 10 min at 80 °C. Next, OsO_4_ was applied for 2 h, followed by washing in cacodylate buffer plus sucrose two times for 5 min. Subsequently, the sample was contrasted with uranyl acetate for 1 h. The specimen was then put into ethanol baths with rising ethanol concentrations for 5 min each, followed by methyl-*tert*-butylether twice for 5 min each, methyl-*tert*-butylether plus epoxide mixture (in 1:3 dilution). Afterwards, the specimens were embedded in an epoxide mixture at 60 °C for 48 h and then at 100 °C for 11.5 h. Semithin and ultrathin sections were cut on a Reichert Jung Ultracut E701704 microtome. Grids were purchased from Polyscience. The grids were then analysed using EM instruments (EM 109 and EM 902, Zeiss) equipped with digital EM cameras (Tröndle). One glomerulus from each case was analysed.

### STED microscopy

Fluorescent labelling of the STED samples was performed as described above. The images were taken with an Abberior Instruments FACILITY LINE microscope equipped with an inverted IX83 microscope (Olympus), a ×60 oil objective (UPlanXApo ×60/1.42 oil, Olympus), using pulsed excitation lasers at 488, 561 and 640 nm and a pulsed STED laser operating at 775 nm, as well as an Abberior Expert Line four-channel easy3D STED instrument equipped with a 775 nm depletion beam (Abberior Instruments), a ×60 oil objective (Nikon ×60/1.4) and a QUAD beam scanner using a pulsed 640 nm diode beam, depleted with the 775 nm STED beam and detected with an avalanche photodiode with a front 685 ± 70 nm band-pass filter. All the acquisition operations were controlled by the Lightbox software Version 16.3.16118. Finally, the deconvolution (Richardson–Lucy) technique was used to improve the image quality.

### 3D-printed imaging chamber

NEXTERION coverslips (size, 110.000 × 74.000 ± 0.200 mm; thickness, 0.175 ± 0.020 mm; SCHOTT) were used for the mounting of expanded tissues that were too large to fit into the Ibidi µ-Slide two-well glass-bottom chambers. A custom-built mounting solution, including a frame, elastic cushion and lid for the frame were printed using a 3D printer to carry the NEXTERION coverslips to allow for mounting in the THUNDER Imager 3D Live Cell and 3D Cell Culture (Leica Microsystems).

The frame carries the NEXTERION coverslip allowing for insertion in the THUNDER Imager 3D Live Cell and 3D Cell Culture (Leica Microsystems). The elastic cushion is placed between the NEXTERION coverslip and lid, preventing the cover glass from breaking when it is locked in place by the lid. The lid for the frame stabilizes the NEXTERION coverslip to the frame. The NEXTERION coverslip and cushion are fully locked to the frame by setting those wedges into notches in the frame. Tinkercad (Autodesk; https://www.tinkercad.com) was utilized to create designs for all the 3D-printed parts.

All 3D-printed parts and design features are shown in Extended Data Figs. 2 and 3. The frame and lid were printed using PolyLite PLA filament 1.75 mm (Polymaker). The cushion was printed using NinjaFlex TPU filament 1.75 mm (NinjaTek). Ender-5 Plus (Creality) was utilized for 3D printing. The settings used in Ultimaker Cura (v. 4.13.1; Ultimaker) are as follows. Nozzle size, 0.40 mm; layer height, 0.16 mm (PLA) and 0.20 mm (TPU); wall thickness, 1.20 mm (PLA) and 0.80 mm (TPU); top/bottom thickness, 1.20 mm (PLA) and 0.80 mm (TPU); nozzle temperature, 220 °C (PLA) and 235 °C (TPU); bed temperature, 50 °C; fan speed, 100%; print speed, 50 mm s^–1^ (PLA) and 20 mm s^–1^ (TPU); first-layer print speed, 25 mm s^–1^ (PLA) and 10 mm s^–1^ (TPU); infil, 10% and zigzag; build-plate adhesion, none. Masking tapes were used to create an adhesive surface on the bed.

### Statistical analyses

All the statistical analyses were performed using GraphPad Prism (v. 9.3.1). Violin plots report the median and interquartile range. Significance was evaluated using the unpaired *t*-tests with Welch’s correction comparing two continuous variables; a paired *t*-test for before/after settings; and the Brown–Forsythe, Welch ANOVA and Dunnett’s tests when comparing three continuous variables. Receiver operating characteristic curves were generated using the Wilson/Brown method (95% confidence interval; results expressed as percentages). A *p* value below 0.05 is considered to be statistically significant.

## Online content

Any methods, additional references, Nature Portfolio reporting summaries, source data, extended data, supplementary information, acknowledgements, peer review information; details of author contributions and competing interests; and statements of data and code availability are available at 10.1038/s41565-023-01328-z.

## Supplementary information

Supplementary Information Supplementary Figs. 1–3.

## Data Availability

All the design files for the 3D-printed imaging chambers are available via GitHub at https://github.com/VPuelleslab/ExSRRF. Source data are deposited at the University Hamburg Research Data Repository at https://www.fdr.uni-hamburg.de/ with the following identifier: 10.25592/uhhfdm.11224.

## Code availability

All code is available via GitHub at https://github.com/imsb-uke/exsrrf_analyses.
