# Supplementary material for: Expansion-enhanced super-resolution radial fluctuations enable nanoscale molecular profiling of pathology specimens
Source: Nat Nanotechnol. 2023 Apr 10;18(4):336–42. doi: 10.1038/s41565-023-01328-z (PMC10115634; doi:10.1038/s41565-023-01328-z)
Supplement: Supplementary file 1 — Supplementary Figs. 1–3. [file 41565_2023_1328_MOESM1_ESM.pdf]

nature nanotechnology

Letter https://doi.org/10.1038/s41565-023-01328-z

Expansion-enhanced super-resolution radial fluctuations enable nanoscale molecular profiling of pathology specimens

In the format provided by the authors and unedited

Suppl. Fig. 1

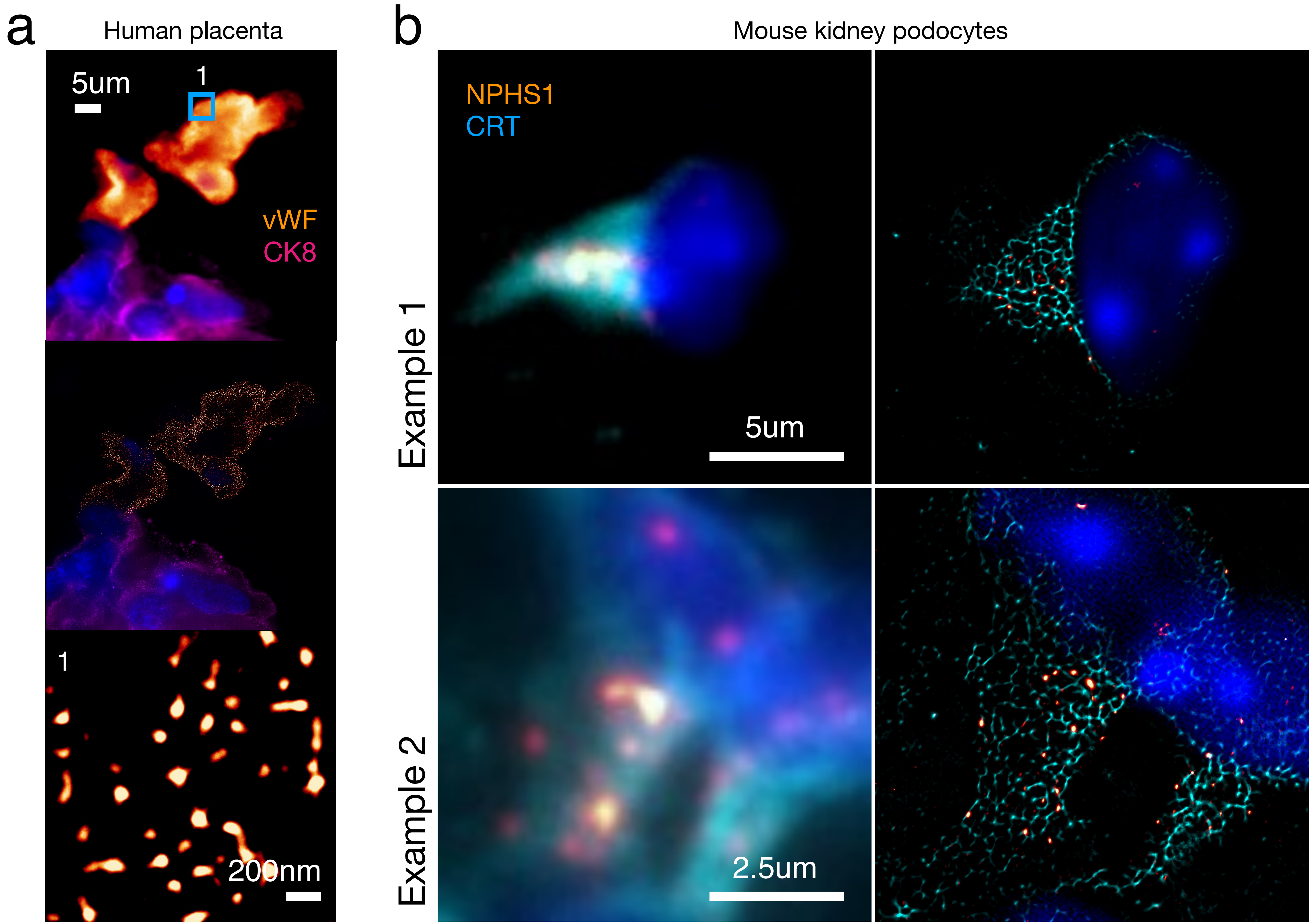

Legend: Additional examples of ExSRRF applications. (a) Even in friable organs, ExSRRF resolves von Willebrand granules, which are imperceptible using widefield microscopy. (b) ExSRRF resolves subcellular location of NPHS1 mRNA (fluorescent in situ hybridization) in calreticulin-based endoplasmic reticulum networks (subsequent indirect immunofluorescence) in murine glomerular podocytes.

Suppl. Fig. 2

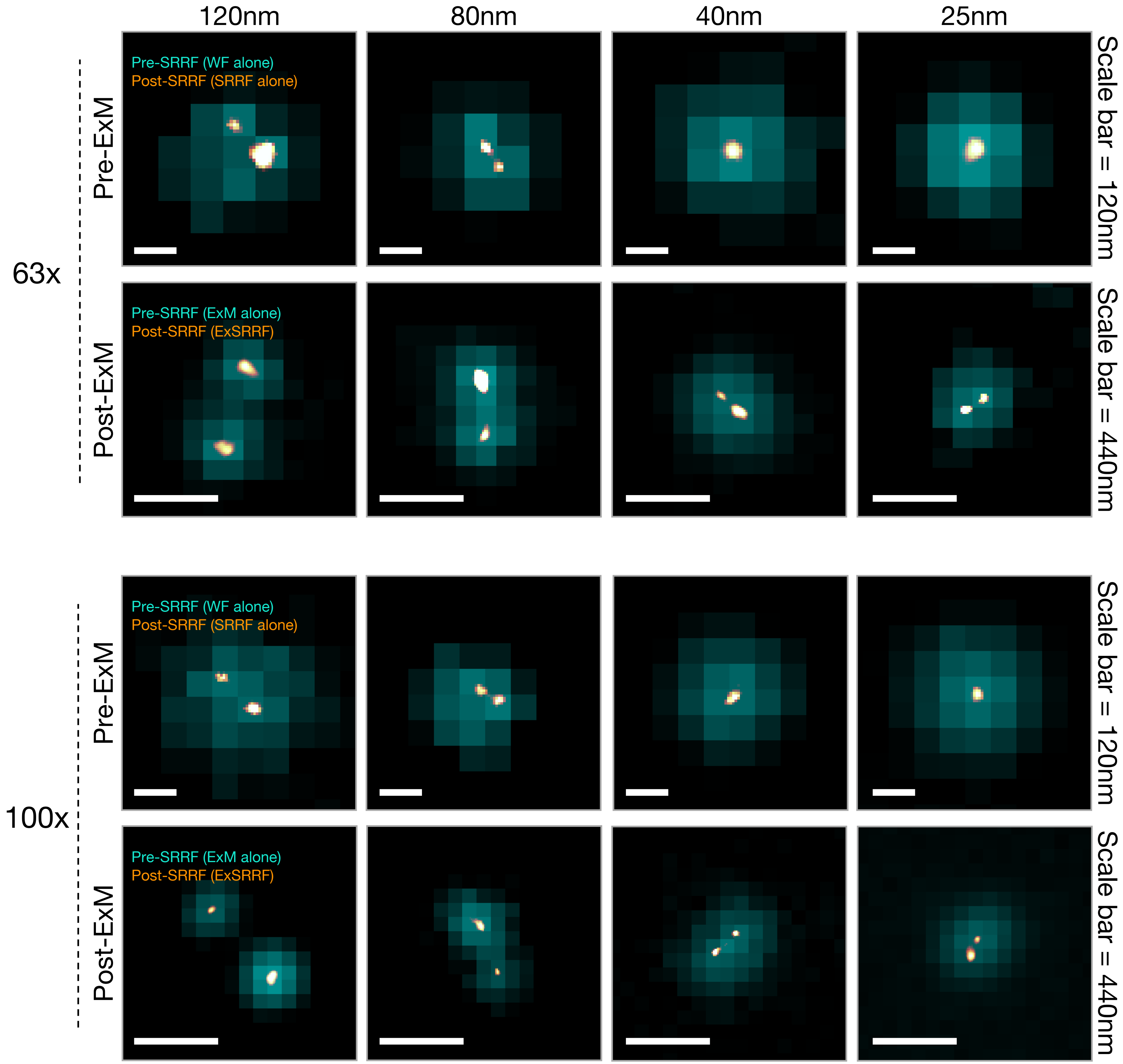

Legend: Validation of nanoruler identification using 63x and 100x objectives in the same LED-based microscopy system. Here we show identical visualization of all used nanorulers using different objectives, which have different numerical apertures and thereby different resolutions.

Suppl. Fig. 3

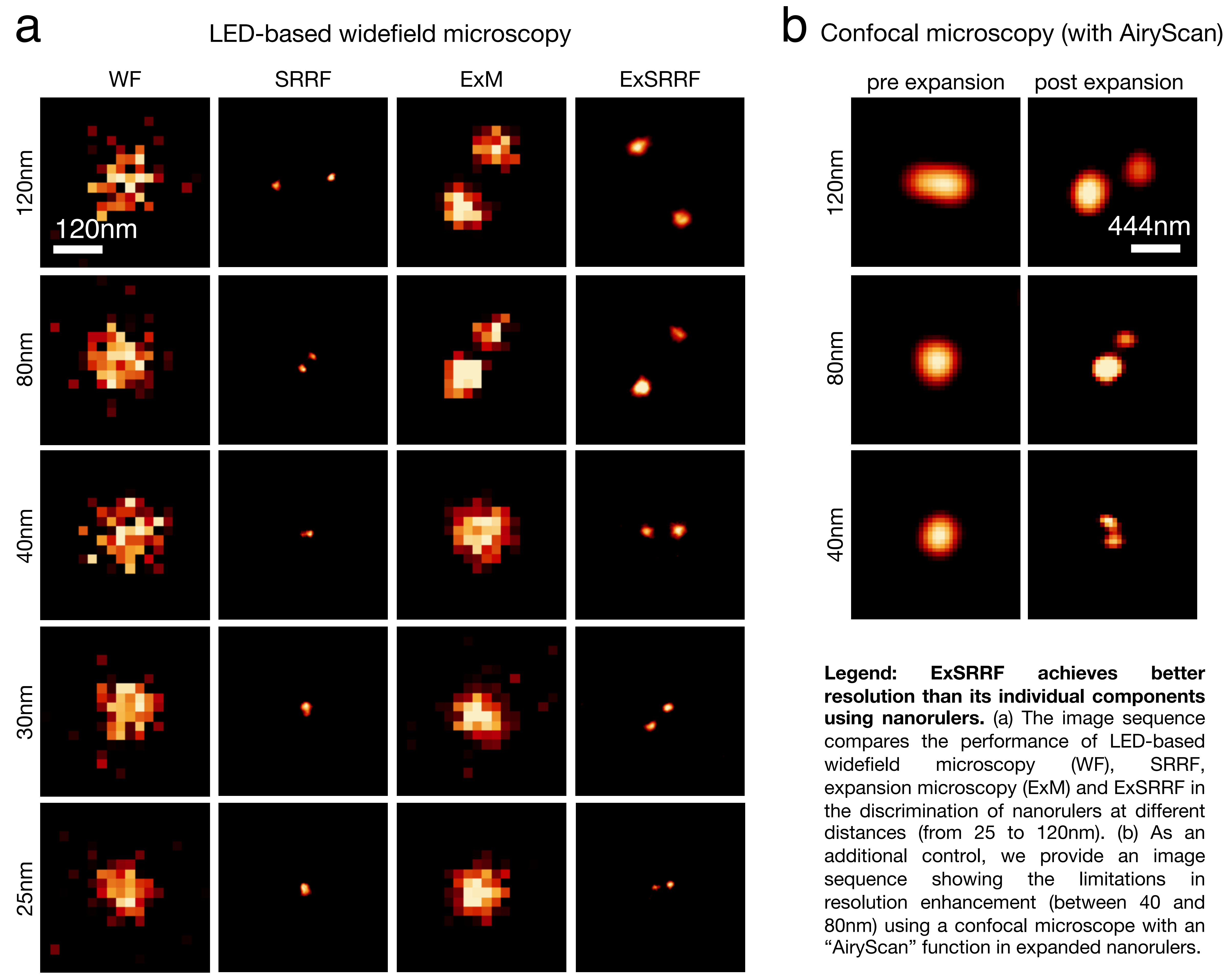

Legend: ExSRRF achieves better resolution than its individual components using nanorulers. (a) The image sequence compares the performance of LED-based widefield microscopy (WF), SRRF, expansion microscopy (ExM) and ExSRRF in the discrimination of nanorulers at different distances (from 25 to 120nm). (b) As an additional control, we provide an image sequence showing the limitations in resolution enhancement (between 40 and 80nm) using a confocal microscope with an "AiryScan" function in expanded nanorulers.
